# NOC4L coordinates neuronal and pharyngeal arch development by regulating ribosome biogenesis

**DOI:** 10.1093/jmcb/mjaf052

**Published:** 2025-12-08

**Authors:** Tujing Song, Yan Liu, Yunxiang Zhou, Xiaoyu Li, Liang Zhang, Guozhu Ning, Jingjing Zhang

**Affiliations:** Zhanjiang Key Laboratory of Zebrafish Model for Development and Disease, Affiliated Hospital of Guangdong Medical University, Zhanjiang 524001, China; School of Medical Technology, Guangdong Medical University, Dongguan 523808, China; Zhanjiang Key Laboratory of Zebrafish Model for Development and Disease, Affiliated Hospital of Guangdong Medical University, Zhanjiang 524001, China; Zhanjiang Key Laboratory of Zebrafish Model for Development and Disease, Affiliated Hospital of Guangdong Medical University, Zhanjiang 524001, China; Zhanjiang Key Laboratory of Zebrafish Model for Development and Disease, Affiliated Hospital of Guangdong Medical University, Zhanjiang 524001, China; Research Center of Translational Medicine, Jinan Central Hospital Affiliated to Shandong First Medical University, Jinan 250013, China; Zhanjiang Key Laboratory of Zebrafish Model for Development and Disease, Affiliated Hospital of Guangdong Medical University, Zhanjiang 524001, China; Zhanjiang Key Laboratory of Zebrafish Model for Development and Disease, Affiliated Hospital of Guangdong Medical University, Zhanjiang 524001, China; School of Medical Technology, Guangdong Medical University, Dongguan 523808, China

**Keywords:** *noc4l*, neuron, pharyngeal arches, ribosome assembly, PPAR, zebrafish

## Abstract

Mutations in ribosome biogenesis-related genes or functional defects in ribosomal proteins can lead to a class of autosomal genetic disorders characterized by tissue-specific defects, termed ribosomopathies. NOC4L, a critical factor in ribosome biogenesis, participates in the maturation of the 40S small ribosomal subunit. However, its functions in neural and cartilage development remain incompletely understood. In this study, through generation and phenotypic characterization of a zebrafish *noc4l* knockout model, we identified severe developmental abnormalities including microcephaly, micrognathia, and embryonic lethality. Further analyses revealed that *noc4l* loss-of-function results in reduced proliferation, differentiation blockade, and apoptotic activation. Mechanistically, sucrose gradient analysis demonstrated the disrupted ribosome biogenesis in *noc4l* mutants, with significantly reduced 40S/80S subunits and polysome levels, ultimately leading to overall translational inhibition and concurrent suppression of metabolic pathways. Pharmacological PPARγ activation via rosiglitazone partially rescued craniofacial malformations, ameliorated neurodevelopmental defects, and prolonged mutant life span. Although inhibition of the p53 pathway can partially rescue the phenotype, the p53 pathway and metabolic pathways are likely independent contributing factors. Our study reveals the molecular basis of developmental defects in *noc4l* mutants through impaired ribosome assembly and demonstrates the therapeutic potential of metabolic interventions for ribosomopathies.

## Introduction

Ribosomopathies refer to a group of autosomal genetic disorders caused by mutations in ribosome biogenesis factors or ribosomal proteins, characterized by tissue-specific phenotypic defects (Yelick and Trainor, 2015). These disorders typically present with key clinical features including developmental defects, craniofacial abnormalities, hematopoietic dysfunction, and an increased risk of cancer (De Keersmaecker et al., 2015; Farley-Barnes et al., 2019; Sulima et al., 2019; Kampen et al., 2020). Although multiple pathogenic mutations in ribosomal proteins and ribosome biogenesis factors have been identified, the molecular mechanisms underlying their pathogenesis remain incompletely elucidated, and therapeutic options for these conditions remain limited.

NOC4L, the human ortholog of yeast Noc4p, functions as an essential ribosome biogenesis factor mediating nuclear export of the 40S ribosomal subunit (Milkereit et al., 2003). Studies have demonstrated that NOC4L deficiency selectively downregulates the expression of mRNAs associated with regulatory T cell activation (Zhu et al., 2019). A recent investigation revealed that NOC4L exerts p53-dependent suppression of tumor cell proliferation and inhibits tumor growth in nude mouse xenograft tumor models (Jia et al., 2022). Additionally, NOC4L has been shown to inhibit TLR4 endocytosis and abrogate the TRIF-dependent signaling pathway, thereby ameliorating chronic low-grade systemic inflammation and insulin resistance in murine models (Qin et al., 2021). Foundational studies demonstrated that targeted disruption of *noc4l* results in preimplantation embryonic lethality in mice (Qin et al., 2017). Notably, the pre-ribosomal factors NOP14, NOC4L, and UTP14A were identified as components of an EMG1-containing nucleolar subcomplex (Warda et al., 2016). This subcomplex is required for the nucleolar recruitment of EMG1. Collectively, these four nucleolar subcomplex members depend on UTP3 for their nucleolar localization (Bao et al., 2024). Nevertheless, the role of NOC4L in organogenesis, particularly its mechanistic involvement in regulating neuronal and skeletal development, remains poorly characterized.

Peroxisome proliferator-activated receptor gamma (PPARγ) functions as a ligand-activated transcription factor within the nuclear receptor superfamily (Mangelsdorf et al., 1995). It encodes two isoforms (PPARγ1 and PPARγ2), generated via alternative splicing (Zhu et al., 1995), and plays pleiotropic roles in lipid metabolism, apoptosis, and inflammation (Sauer, 2015). In the central nervous system (CNS), PPARγ is widely expressed across neuronal, astrocytic, oligodendrocytic, and microglial populations (Bernardo and Minghetti, 2008). Agonists of PPARγ mitigate neuroinflammation in Parkinson’s disease models by suppressing microglial and astrocytic activation; for instance, rosiglitazone reduces dopaminergic neuron loss (Pisanu et al., 2014). Downregulation of PPARγ impairs neural progenitor differentiation (Di Giacomo et al., 2017), while its upregulation counteracts drug-induced brain developmental defects (Muhsen et al., 2021). In cartilage development, PPARγ expression is indispensable for normal cartilage morphogenesis (Monemdjou et al., 2012), and its inhibition promotes chondrocyte apoptosis (Ji et al., 2020), highlighting its key role in chondrocyte survival and function. The involvement of PPARγ in ribosomopathies remains unclear.

In this study, we utilized CRISPR/Cas9 gene knockout technology to generate *noc4l* homozygous mutant zebrafish and found that *noc4l* mutation exhibited embryonic lethality at early developmental stages, accompanied by characteristic phenotypes including microcephaly and micrognathia. Using this zebrafish model, we investigated the molecular mechanisms underlying the head and pharyngeal arch developmental defects caused by *noc4l* deficiency and identified potential therapeutic strategies that may alleviate microcephaly and micrognathia in patients.

## Results

### noc4l exhibits specific high expression in the nervous system and pharyngeal arches of zebrafish embryos

**Figure 1:**
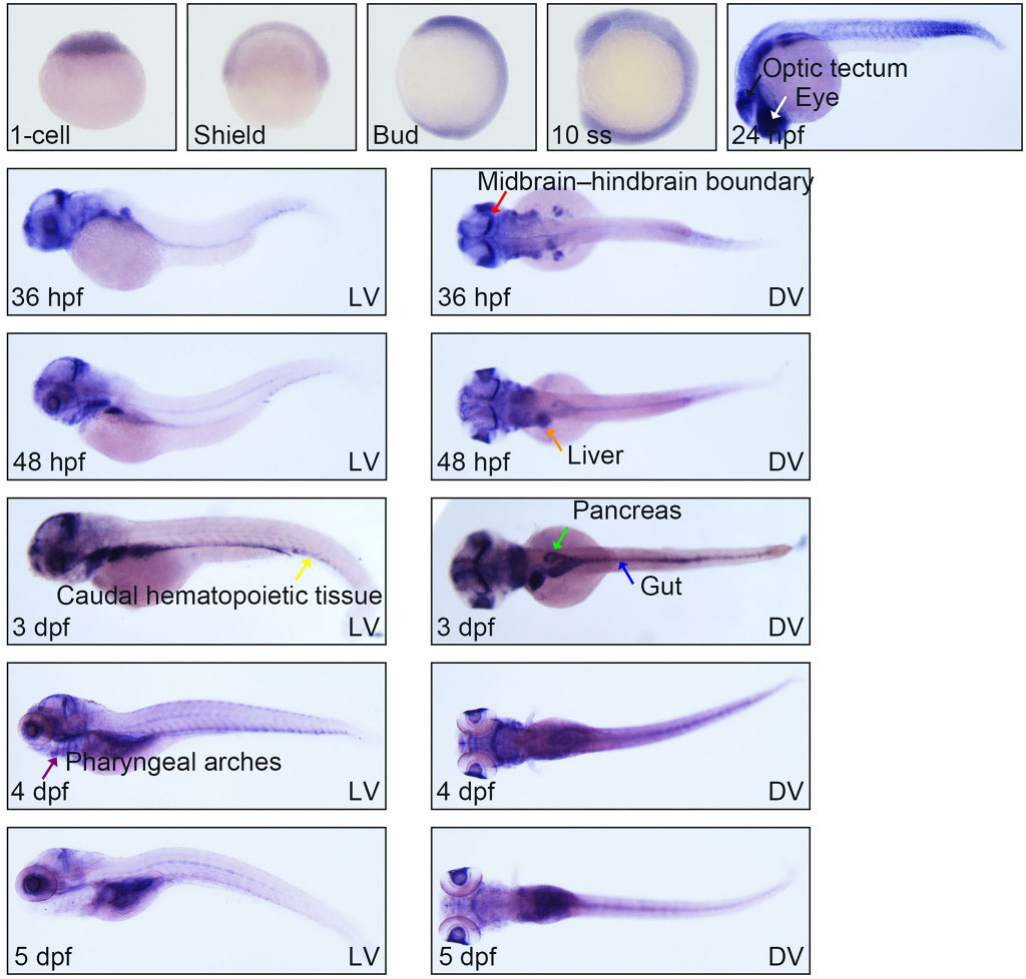
Expression patterns of *noc4l* in early zebrafish development. WISH analysis reveals the spatial and temporal expression of *noc4l* at the indicated developmental stages: 1-cell, shield, bud, 10 ss, 24 hpf, 36 hpf, 48 hpf, 3 dpf, 4 dpf, and 5 dpf. Arrows highlight specific anatomical structures: optic tectum (black), eye (white), midbrain–hindbrain boundary (red), liver (orange), CHT (yellow), pancreas (green), gut (blue), and pharyngeal arches (purple). Scale bar, 100 μm. ss, somite stage; LV, lateral view; DV, dorsal view.

This study focuses on NOC4L, a member of the nucleolar subcomplex. Leveraging the advantages of the zebrafish model, to explore NOC4L’s functional roles and biological significance in developmental biology, we first systematically analyzed the spatiotemporal expression patterns of the *noc4l* gene during zebrafish embryonic development. Whole-mount *in situ* hybridization (WISH) results showed that *noc4l* transcripts were expressed from the zygotic stage (Figure 1). By the end of gastrulation, *noc4l* exhibited a broad expression pattern (Figure 1). As embryonic development progressed, by 24 hours post-fertilization (hpf), the expression of *noc4l* expanded to structures such as the retina, optic tectum, muscle, and endoderm (Figure 1). After 36 hpf, the expression of *noc4l* gradually became tissue-specific, mainly confined to organs including the midbrain–hindbrain boundary, eyes, pharyngeal arches, digestive system, and caudal hematopoietic tissue (CHT) (Figure 1). It exhibited high expression consistency with the nucleolar subcomplex members *nop14* and *emg1* (Supplementary Figure S1). Notably, specific and elevated expression of *noc4l* was observed in the nervous system and pharyngeal arches of zebrafish embryos.

### Loss of noc4l function in zebrafish results in developmental abnormalities

Given the prominent expression of *noc4l* in the midbrain–hindbrain boundary and pharyngeal arches, we utilized CRISPR/Cas9 gene-editing technology to generate a *noc4l* mutant zebrafish strain. Specifically, a single-guide RNA (gRNA) that targets the fourth exon of the *noc4l* gene was co-injected with Cas9 protein into wild-type embryos at the single-cell stage, successfully introducing a 5-base-pair deletion in the coding sequence of *noc4l* (Supplementary Figure S2A). This deletion caused premature termination of Noc4l translation, resulting in a truncated protein of only 185 amino acid residues (Supplementary Figure S2A). To validate the knockout efficiency, WISH analysis was performed at 3.5 days post-fertilization (dpf), revealing a significant reduction in *noc4l* mRNA expression levels in the mutants, confirming the successful generation of the *noc4l* knockout zebrafish strain (Supplementary Figure S2B). Quantitative real-time PCR (Q-PCR) analysis revealed a significant downregulation of *noc4l* mRNA expression in *noc4l* mutants (Supplementary Figure S2C). In mouse models, homozygous *noc4l* mutations are embryonically lethal. However, in zebrafish, homozygous *noc4l* mutants can survive until 5–12 dpf, likely due to the maternally deposited *noc4l*, which sustains zebrafish embryogenesis in the absence of zygotic *noc4l*.

**Figure 2:**
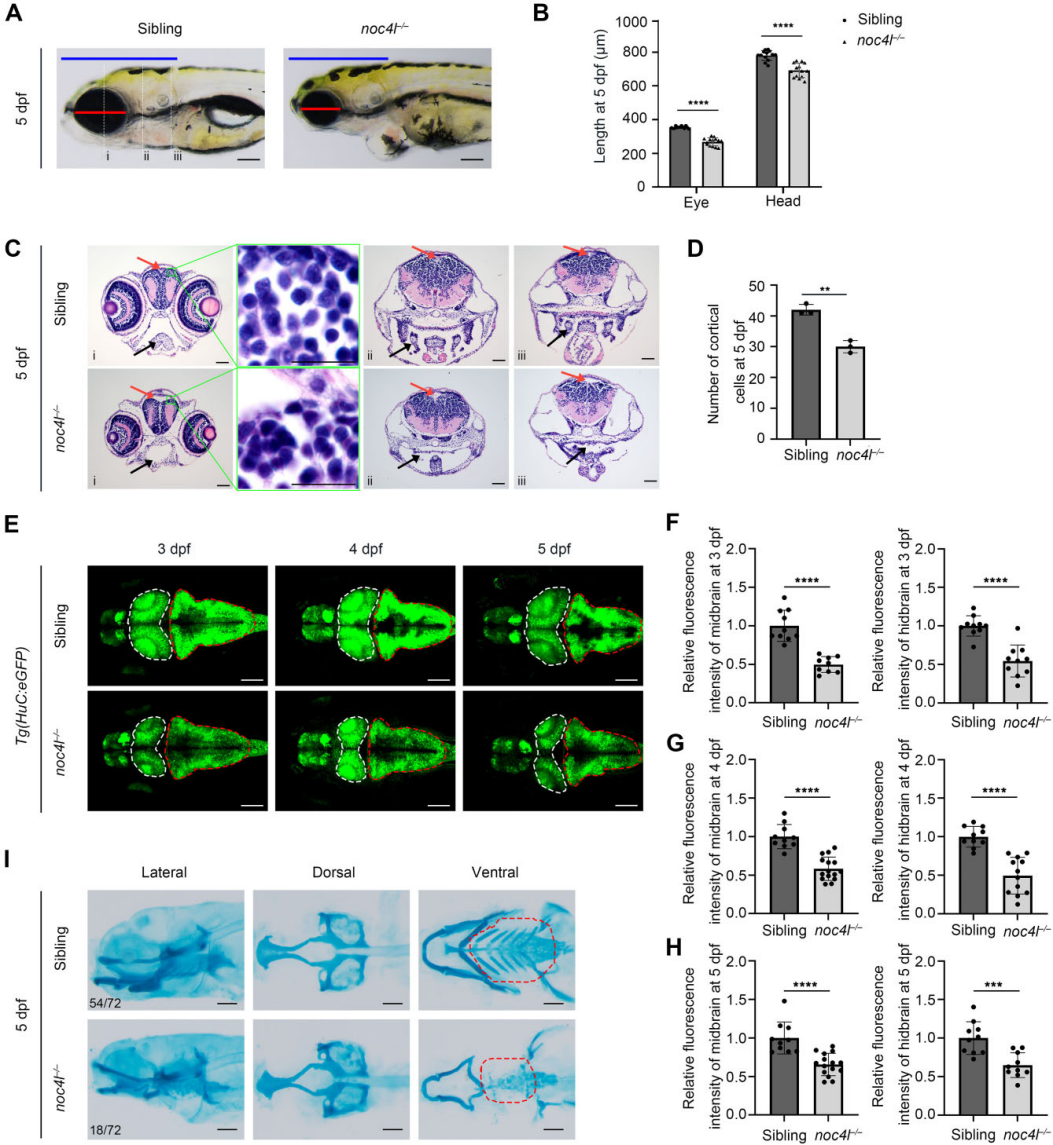
Phenotypic characterization of *noc4l* mutants. (**A**) Bright-field images (lateral view) of 5 dpf siblings and *noc4l* mutants. Mutants display reduced head size (blue line), smaller eyes (red line), diminished mandibular structures, pericardial edema, and absent swim bladder. (**B**) Quantification of head size and eye size at 5 dpf (*n* > 3, ****P < 0.0001). (**C**) HE staining of transverse paraffin sections (locations indicated by white dotted lines in **A**) of the brain at 5 dpf. Black arrows: mandible; red arrows: brain ventricle. (**D**) Quantification of cortical cell numbers in the region marked in **C** (*n* = 3 biologically independent animals, mean ± SD, *P* < 0.01). (**E**) Dorsal views of neuronal expression in the *Tg(HuC:eGFP)* background at 3 dpf, 4 dpf, and 5 dpf. White and red dotted circles indicate midbrain and hindbrain regions, respectively. (**F**–**H**) Quantification of relative fluorescence intensity in the midbrain and hindbrain at 3 dpf (**F**; *n* > 3, ****P < 0.0001), 4 dpf (**G**; *n* > 3, ****P < 0.0001), and 5 dpf (**H**; midbrain: *n* > 3, ****P < 0.0001; hindbrain: *n* > 3, ***P < 0.001). (**I**) Alcian blue staining of the head region (lateral, dorsal, and ventral views) at 5 dpf, showing malformed pharyngeal arch development. Red dotted circles indicate the ceratobranchial cartilage. Scale bar, 100 μm.

We compared morphologies of *noc4l* mutants and sibling controls at 72 hpf, 3.5 dpf, and 4 dpf and measured their head length and eye width. At 72 hpf, *noc4l* mutants showed no significant difference in head morphology. However, after 72 hpf, *noc4l* mutants exhibited smaller heads, eyes, and pharyngeal arches compared to their siblings (Supplementary Figure S3A and B). *noc4l* mutants showed progressive lethality from 5 dpf, and the last surviving mutants died at 12 dpf. By 5 dpf, developmental defects in *noc4l* mutants aggravated, with significantly smaller heads and eyes compared to siblings (Figure 2A and B). Additionally, *noc4l* mutants exhibited marked cartilage developmental defects, along with pericardial edema and absent swim bladders (Figure 2A). To further characterize the phenotypes, we measured the body lengths of sibling controls and *noc4l* mutants at 5 dpf. No significant difference was observed between two groups (Supplementary Figure S3C and D), suggesting that the aforementioned phenotypic defects are specific and not attributable to systemic developmental delay. Notably, the *noc4l* mutants with the 1-base-pair deletion (with a 15-bp insertion) genotype exhibited consistent phenotypes (Supplementary Figure S4). This study selected the *noc4l* mutants with a 5-base-pair deletion genotype for subsequent phenotypic analysis and mechanistic investigation. In order to ensure genotype consistency across samples in subsequent experiments, the embryos were genotyped by PCR as shown in Supplementary Figure S2D.

To investigate the internal structural changes in *noc4l* mutants, we performed paraffin sectioning and hematoxylin–eosin (HE) staining on zebrafish samples. Cross-section analysis of three distinct planes (Figure 2A) corresponding to midbrain and hindbrain regions revealed significant cerebral ventricular dilation and mandibular cartilage absence in all planes of *noc4l* mutants (Figure 2C). Given the association between microcephaly and the reduced cortical neuron number, we quantified cortical neurons in the first plane and found a significant decrease in *noc4l* mutants (Figure 2C and D).

High expression level of *noc4l* in zebrafish brain and pharyngeal arch regions during development suggests that *noc4l* is essential for neural tissue and pharyngeal arch development. To test this hypothesis, we crossed *noc4l* mutants with a neuron-specific reporter *HuC:eGFP* zebrafish line (Moon et al., 2020). Fluorescence reporter analysis revealed that the relative fluorescence intensity of neurons in the midbrain and hindbrain regions of *noc4l* mutants was significantly lower than that of siblings at 3 dpf, 4 dpf, and 5 dpf (Figure 2E), indicating a marked reduction in the number of neurons in these regions of the mutants. Statistical analysis confirmed the significance of these differences (Figure 2F–H). Alcian blue staining showed that most *noc4l* mutants had abnormal pharyngeal arch structures at 5 dpf, with the reduced ceratobranchial cartilage number in pharyngeal arches (Figure 2I). Importantly, injection of 200 pg *noc4l* mRNA was able to restore both defects (Supplementary Figure S5). In addition, we found that the liver size of *noc4l* mutant zebrafish was smaller at 3.5 dpf (Supplementary Figure S6A). Meanwhile, the expression of the hematopoietic stem cell marker *cmyb* slightly decreased and the expression of the T-cell marker *rag1* was abolished in the thymus of *noc4l* mutants at 4 dpf (Supplementary Figure S6B). However, these hepatic and hematopoietic developmental defects were not life-threatening. Altogether, *noc4l* mutant zebrafish exhibited developmental defects in cranial neurons and pharyngeal arches.

### Impaired differentiation, decreased proliferation, and increased apoptosis contribute to brain and pharyngeal arch defects in noc4l mutants

**Figure 3:**
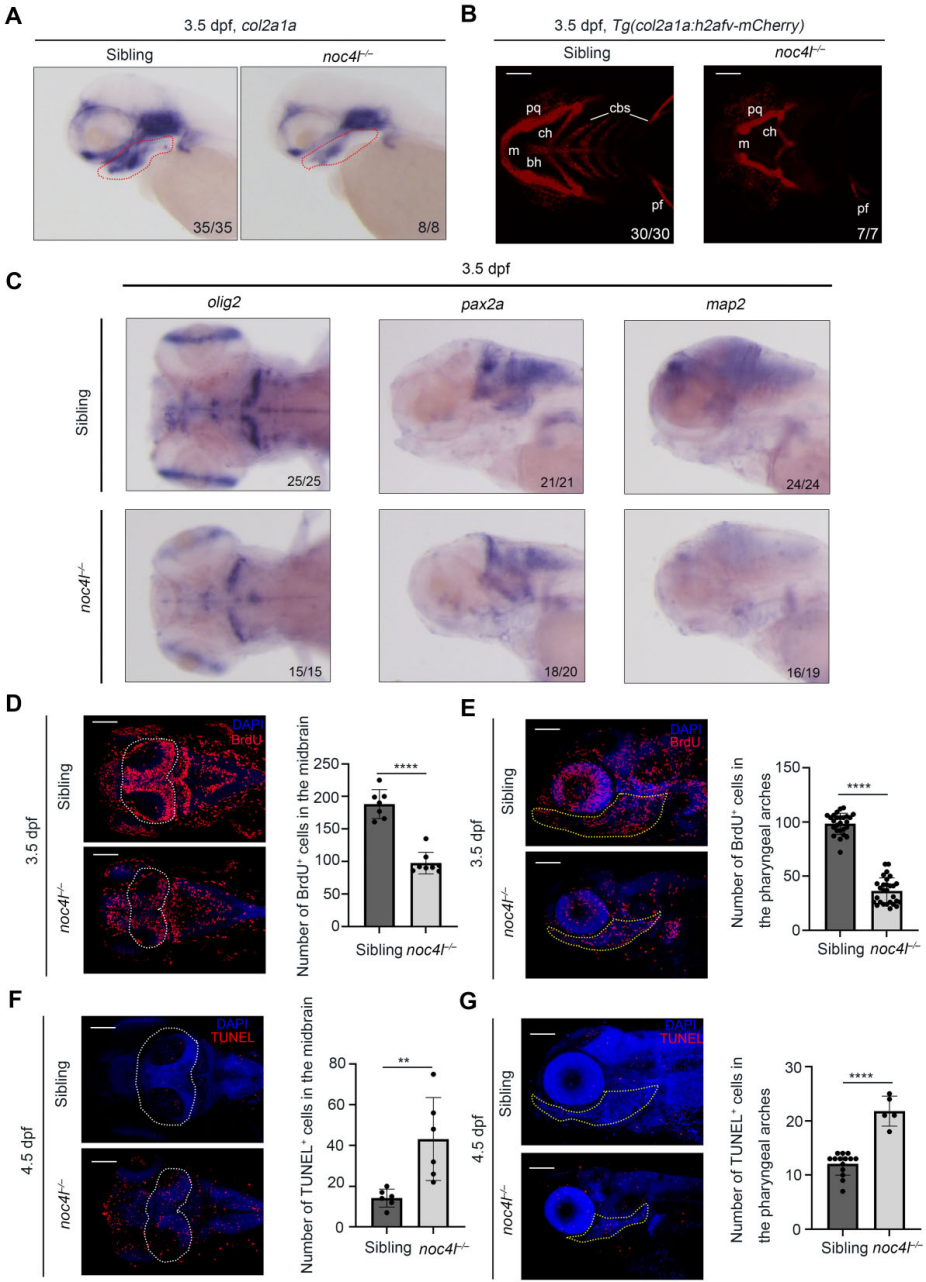
Analyses of cell differentiation, proliferation, and apoptosis in the midbrain and pharyngeal arches of siblings and *noc4l* mutants. (**A**) Altered expression of *col2a1a* in 3.5 dpf *noc4l* mutants compared to siblings. The dashed line outlines the pharyngeal area. (**B**) Ventral views of the head region in *Tg(col2a1a:h2afv-mCherry)* larvae at 3.5 dpf, revealing cartilage developmental defects in mutants. bh, basihyal; cb, ceratobranchial; ch, ceratohyal; m, Meckel's cartilage; pq, palatoquadrate; pf, pectoral fins. (**C**) Expression changes of neuronal markers (*olig2, pax2a*, and *map2*) in 3.5 dpf *noc4l* mutants. (**D**) BrdU immunofluorescence (red) detecting cell proliferation in the midbrain (dorsal view; white dotted circle). Right panel: quantification of BrdU-positive cells in the midbrain (*n* > 3, ****P < 0.0001). (**E**) BrdU immunofluorescence (red) detecting cell proliferation in the pharyngeal arches (lateral view; yellow dotted circle). Right panel: quantification of BrdU-positive cells in the pharyngeal arches (*n* > 3, ****P < 0.0001). (**F**) TUNEL assay (red) detecting apoptosis in the midbrain (dorsal view; white dotted circle). Right panel: quantification of TUNEL-positive cells in the midbrain (*n* > 3; **P < 0.01). (**G**) TUNEL assay (red) detecting apoptosis in the pharyngeal arches (lateral view; yellow dotted circle). Right panel: quantification of TUNEL-positive cells in the pharyngeal arches (*n* > 3; ****P < 0.0001). Scale bar, 100 μm.

Morphological analysis indicated that the developmental defects in the brain and pharyngeal arches of *noc4l* mutants might be associated with changes in cell number. Then, we examined the levels of cell differentiation, proliferation, and apoptosis within the brain and pharyngeal arch regions of *noc4l* mutants and their siblings at 3.5 dpf or 4.5 dpf. Expression level of the differentiating chondrocyte marker *col2a1a* (Yan et al., 2002) was reduced in the ceratobranchial cartilage of *noc4l* mutants at 3.5 dpf (Figure 3A). The ceratobranchial cartilage disappeared in the *Tg(col2a1a:h2afv-mCherry)* transgenic background (Figure 3B), confirming cartilage developmental defects in *noc4l* mutants. Furthermore, we analyzed the expression profiling of the oligodendrocyte marker *olig2* (Zaucker et al., 2013), the CNS marker *map2*, and the midbrain–hindbrain boundary marker *pax2a* (Lei et al., 2017). All these markers showed the reduced expression in the brain of *noc4l* mutants at 3.5 dpf, indicating that *noc4l* knockout leads to impaired differentiation of neurons and pharyngeal cartilage (Figure 3C). Immunofluorescence results revealed a significant reduction in bromodeoxyuridine (BrdU) signal in the midbrain and pharyngeal arch regions of *noc4l* mutants, indicating decreased cell proliferation (Figure 3D and E). Additionally, TUNEL assays detected prominent apoptotic signals in the midbrain and pharyngeal arch regions of *noc4l* mutants, suggesting elevated levels of apoptosis (Figure 3F and G). Collectively, brain and pharyngeal arch defects in *noc4l* mutants were driven by impaired cell differentiation, reduced proliferation, and elevated apoptosis.

### Ribosome synthesis and protein translation are compromised in noc4l mutants

**Figure 4:**
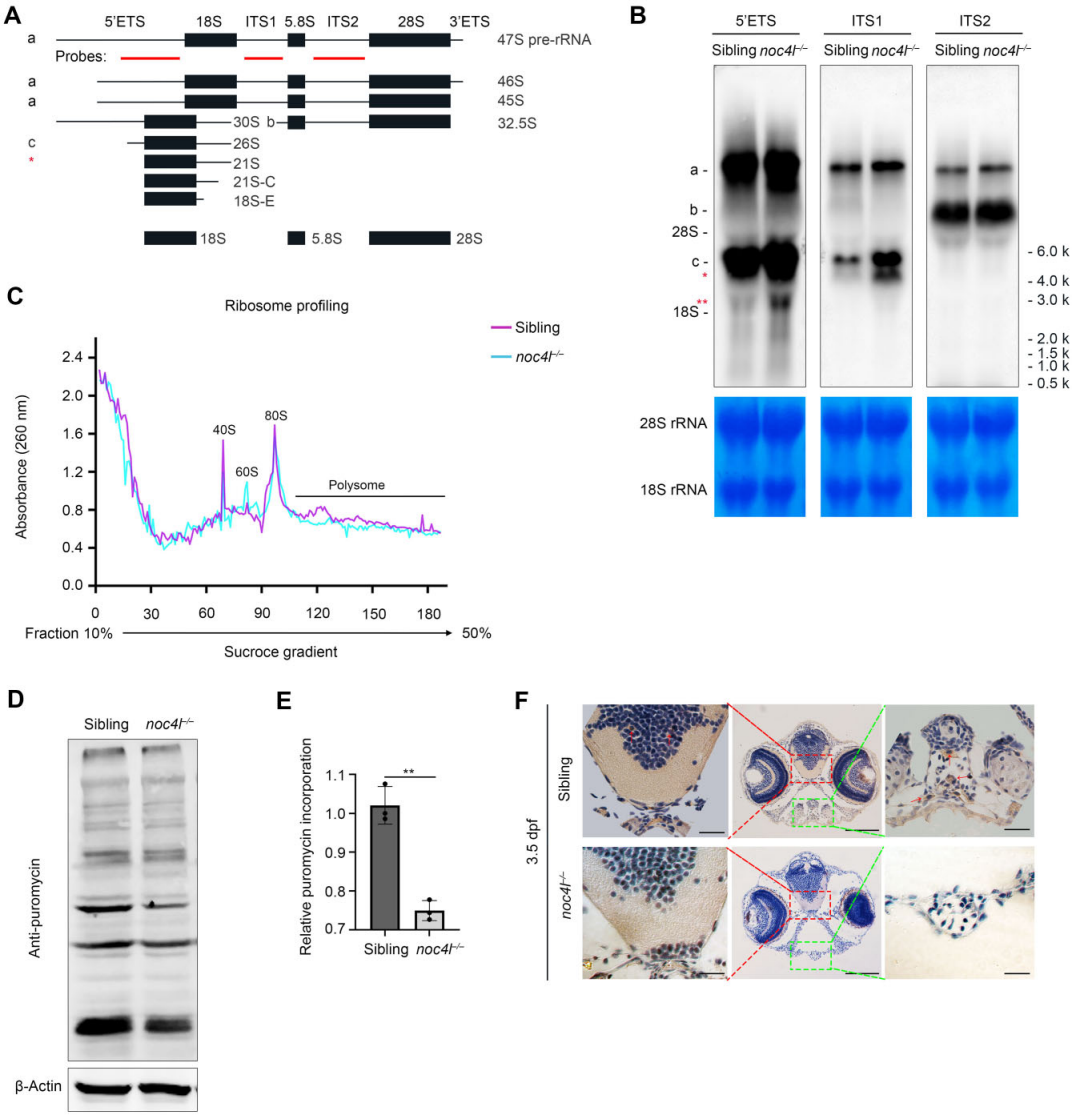
Impairment of rRNA processing and protein translation in *noc4l* mutants. (**A**) Schematic of the 47S pre-rRNA processing pathway in zebrafish, with probe locations (red lines) for 5′ETS, ITS1, and ITS2 indicated. (**B**) Northern blot analysis of 5 dpf siblings and mutants using the specified probes. Bands corresponding to normal 47S pre-rRNA intermediates (a, b, c) and additional intermediates (red asterisks) are marked. Methylene blue staining of 28S and 18S rRNA serves as the loading control. (**C**) Ribosome profiling analyzed by sucrose density gradient centrifugation in 5 dpf siblings and mutants. (**D** and **E**) Puromycin incorporation assay assessing nascent protein synthesis at 3.5 dpf, detected by immunoblotting with an anti-puromycin antibody. β-Actin serves as the loading control. Quantification of band intensity shows an ∼25% reduction in global translation efficiency in mutants (*n* = 3, **P < 0.01). (**F**) IHC on transverse paraffin sections using an anti-puromycin antibody (brown deposition, red arrows) reveals localized translation defects at 3.5 dpf. Scale bar, 100 μm.

According to previous literature, NOC4L is a crucial molecule closely associated with ribosome biogenesis (Milkereit et al., 2003). To determine whether ribosome synthesis as well as protein translation are impaired in *noc4l* mutants, we analyzed the processing of 47S pre-rRNA in siblings and *noc4l* mutants by northern blotting, employing probes that target the 5′ETS, ITS1, and ITS2 areas of 47S pre-rRNA (Figure 4A). No abnormal pre-rRNA processing intermediates were detected by the ITS2 probe, while an additional smaller band was detected by either the 5′ETS or ITS1 probe (Figure 4B), indicating a defect in 18S rRNA processing. Subsequently, we analyzed the ribosome profiling in *noc4l* mutants and siblings at 5 dpf by sucrose density gradient centrifugation. The results revealed a significant reduction in 40S and 80S ribosomal subunits and polysomes but an increase in the 60S ribosomal subunit in *noc4l* mutants (Figure 4C). This defect in ribosome biogenesis further suppressed nascent protein synthesis, evidenced by the results of puromycin incorporation assays (Figure 4D and E) and immunohistochemical (IHC) staining of brain sections with an anti-puromycin antibody (Figure 4F). Taken together, *noc4l* mutants exhibited impaired ribosome synthesis and protein translation.

### Metabolic pathways are downregulated in noc4l mutants

**Figure 5:**
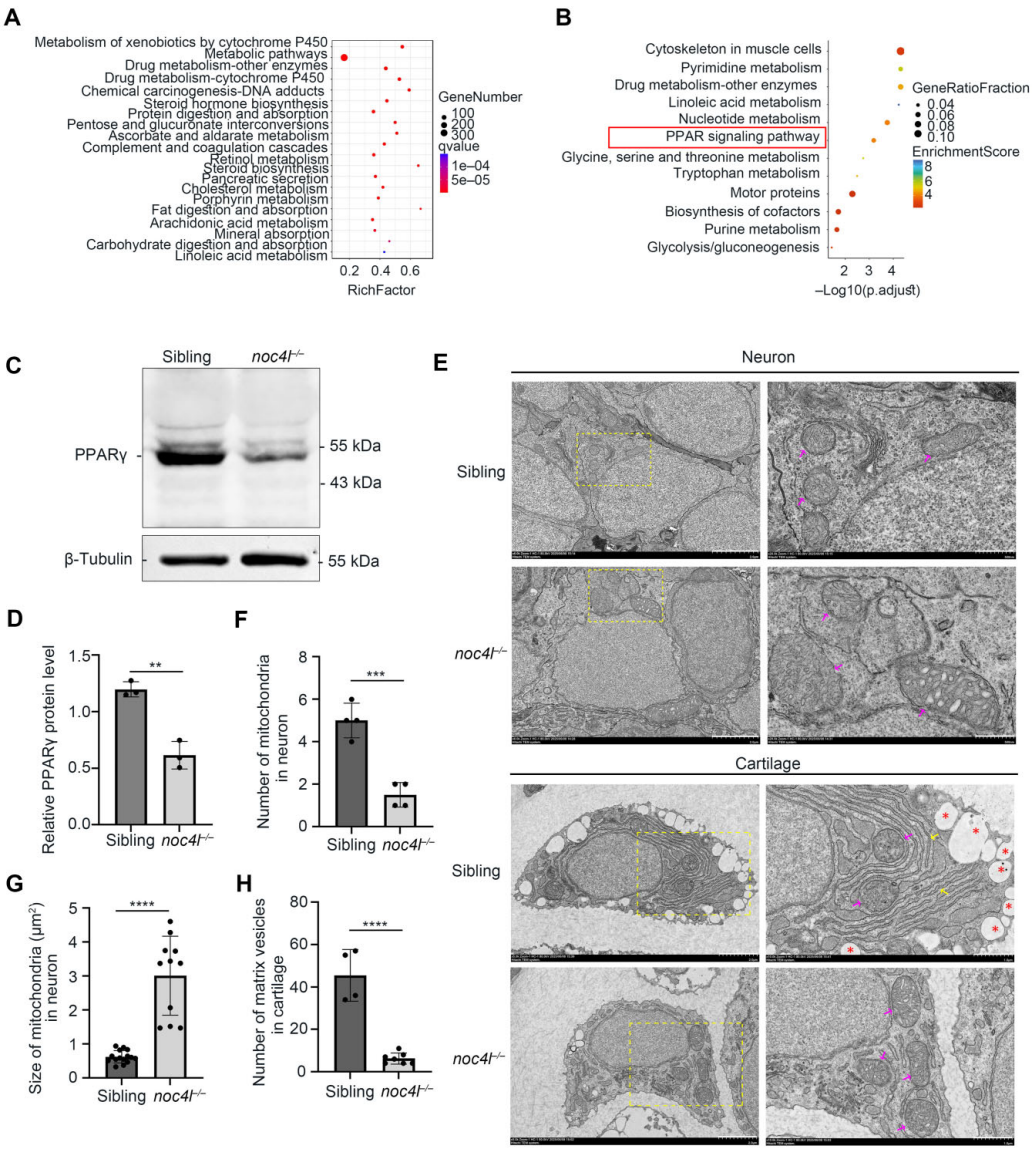
Downregulation of the PPAR pathway in *noc4l* mutants. (**A**) KEGG pathway analysis of DEGs from RNA-seq data of 5 dpf head tissues. (**B**) KEGG pathway analysis of genes with reduced translation efficiency from Ribo-seq data. The PPAR signaling pathway is highlighted (red box). (**C** and **D**) Western blot analysis and quantification of PPARγ protein levels in 5 dpf siblings and mutants (*n* = 3, **P < 0.01, *t*-test). β-Tubulin serves as the loading control. (**E**) TEM images of brain neurons and cartilage cells. Pink arrows: mitochondria; yellow arrows: ER; red stars: matrix vesicles. Right panels show enlargements of the boxed areas. (**F**) Quantification of the number of mitochondria in neuron (*n* > 3, ***P < 0.001). (**G**) Quantification of the size of mitochondria in neuron (*n* > 3, ****P < 0.0001). (**H**) Quantification of the number of matrix vesicles in cartilage cell (*n* > 3, ****P < 0.0001).

To identify differentially expressed genes (DEGs) and elucidate the pathways affected by *noc4l* knockout, we performed RNA sequencing (RNA-seq) analysis. The results showed that the DEGs between *noc4l* mutants and siblings were primarily enriched in metabolic pathways, which are crucial for maintaining basic cellular physiological functions (Figure 5A). To further investigate the impact of ribosome biogenesis defects on downstream signaling pathways in *noc4l* mutants, we conducted a systematic ribosome sequencing (Ribo-seq) analysis. The translation efficiencies for each gene in siblings and *noc4l* mutants at 5 dpf were calculated and compared to identify the genes with significantly reduced translation efficiency in mutants. These genes were primarily enriched in metabolic pathways, in particular a marked downregulation in the PPAR signaling pathway, which is mainly involved in regulating cellular metabolism (Figure 5B). Studies have demonstrated that nuclear receptor PPARγ, a member of the PPAR family, plays a critical role in regulating the development of brain tissue and cartilage (Monemdjou et al., 2012; Muhsen et al., 2021). Consistent with the Ribo-seq results, western blot analysis revealed a significantly lower level of PPARγ protein in *noc4l* mutants (Figure 5C and D). Transmission electron microscopy (TEM) images revealed mitochondrial dysfunction in neurons of *noc4l* mutants, characterized by the mild swelling, decreased number, and increased size of mitochondria (Figure 5E–G), as well as the absence of endoplasmic reticulum (ER) and decreased number of normal matrix vesicles in pharyngeal cartilage (Figure 5E and H). Therefore, *noc4l* deficiency compromised cellular metabolism along with mitochondrial and ER homeostasis.

### Activation of the PPARγ pathway partially rescues brain and pharyngeal arch malformations in noc4l mutants

**Figure 6:**
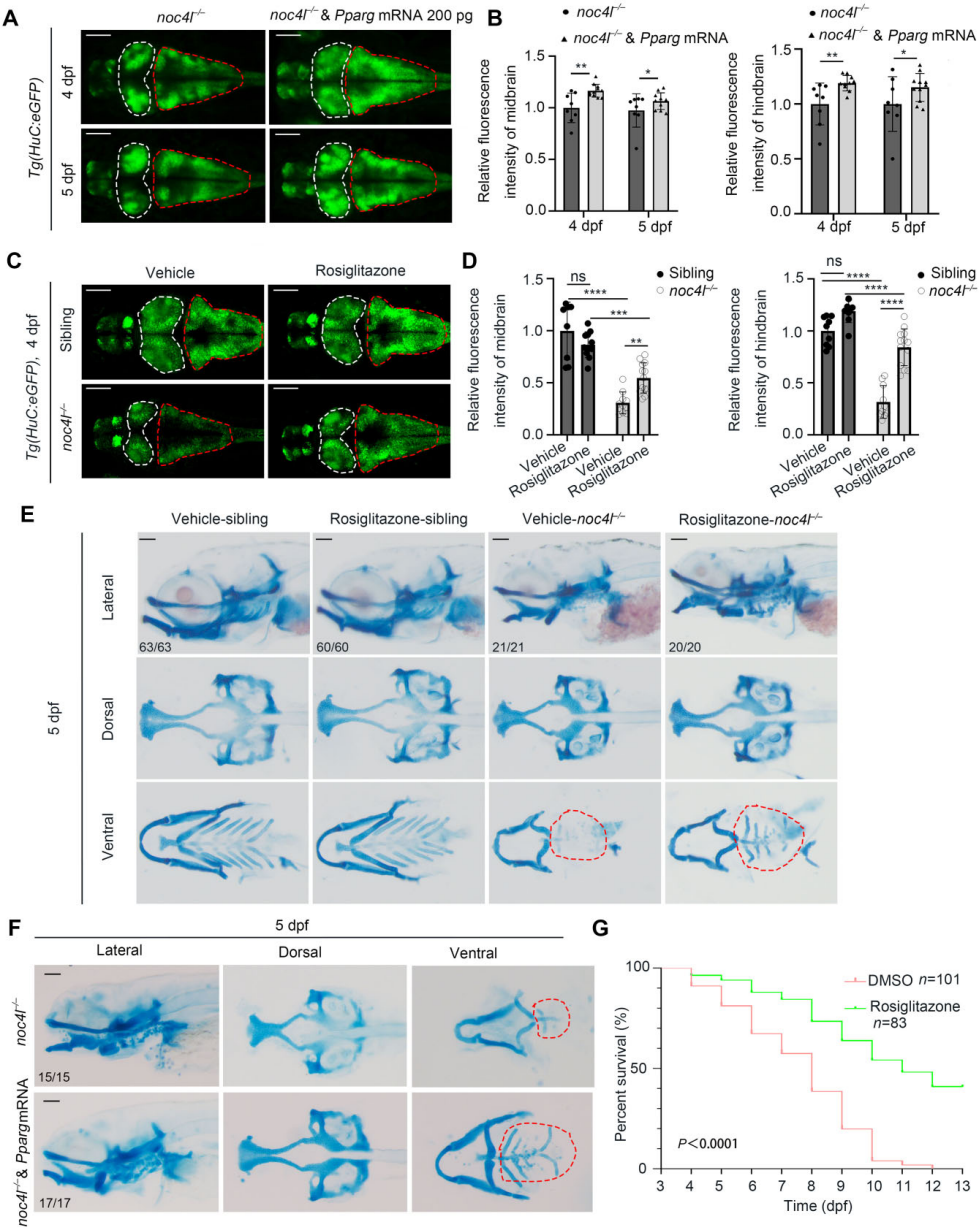
Activation of the PPARγ pathway partially restores brain and pharyngeal arch abnormalities in *noc4l* mutants. (**A**) Neuronal expression in *Tg(HuC:eGFP)* embryos at 4 dpf and 5 dpf following microinjection of *Pparg* mRNA (200 pg/nl, 1 nl/embryo) at the 1-cell stage. White and red dashed circles indicate midbrain and hindbrain regions, respectively. (**B**) Quantification of relative fluorescence intensity in the midbrain and hindbrain at 4 dpf (*n* > 3, **P < 0.01) and 5 dpf (*n* > 3, *P < 0.05). (**C**) Neuronal expression in *Tg(HuC:eGFP)* embryos at 4 dpf after treatment from 2.5 dpf with 1 μM rosiglitazone or DMSO (vehicle control). White and red dotted circles indicate the midbrain and hindbrain, respectively. (**D**) Quantification of relative fluorescence intensity in the midbrain (*n* > 3, ns (*P* > 0.05), **P < 0.01, ***P < 0.001, ****P < 0.0001) and hindbrain (*n* > 3, ns (*P* > 0.05), ****P < 0.0001) at 4 dpf. (**E**) Alcian blue staining of the head region (lateral, dorsal, and ventral views) at 5 dpf after rosiglitazone treatment, showing partial rescue of pharyngeal arch defects. Red dotted circles indicate ceratobranchial cartilage. (**F**) Alcian blue staining of the head region at 5 dpf following *Pparg* mRNA injection, showing phenotypic rescue. (**G**) Kaplan–Meier survival curves of embryos treated from 2.5 dpf with 1 μM rosiglitazone or DMSO (DMSO: *n* = 101; rosiglitazone: *n* = 83). Survival was significantly improved by rosiglitazone treatment (Log-rank test, ****P < 0.0001). The treatment timeline is shown in the inset. Scale bar, 50 μm (**A** and **C**) or 100 μm (**E** and **F**).

Given that PPARγ is a nuclear regulator of metabolism, differentiation, and cell growth (Rosen and Spiegelman, 2001), we hypothesized that these ultrastructural abnormalities may be associated with the impaired PPARγ signaling. Indeed, injection of 200 pg *Pparg* mRNA partially restored neuronal development (Figure 6A and B) and ceratobranchial cartilage formation (Figure 6F). Then, we treated the embryos with the PPARγ agonist, rosiglitazone. Phenotypic analysis showed that at 3.5 dpf, the head size of rosiglitazone-treated *noc4l* mutant embryos was significantly larger than that of dimethyl sulfoxide (DMSO)-treated *noc4l* mutant embryos (Supplementary Figure S7A and B), indicating that activation of the PPARγ pathway partially rescued the brain developmental defects in *noc4l* mutants. Furthermore, we performed drug treatment in *noc4l* mutants with the *Tg(HuC:eGFP)* background and detected neuronal fluorescence intensity. At 4 dpf, the relative fluorescence intensity of neurons in the midbrain and hindbrain regions was significantly higher in rosiglitazone-treated *noc4l* mutants than in DMSO-treated *noc4l* mutants (Figure 6C and D), suggesting that PPARγ pathway activation partially restored neurons in the midbrain and hindbrain regions of the mutants. Additionally, Alcian blue staining analysis of embryos at 5 dpf revealed that the number of ceratobranchial cartilages significantly increased in rosiglitazone-treated *noc4l* mutants compared to DMSO-treated *noc4l* mutants (Figure 6E). The survival rate of embryos increased to 40.96% with the treatment of rosiglitazone, indicating that rosiglitazone treatment can prolong the life span of the mutants (Figure 6G). Overall, malformations in the brain and pharyngeal arches of *noc4l* mutants were partly alleviated by activation of the PPARγ pathway.

### The partial rescue exhibits incomplete p53 dependence

**Figure 7:**
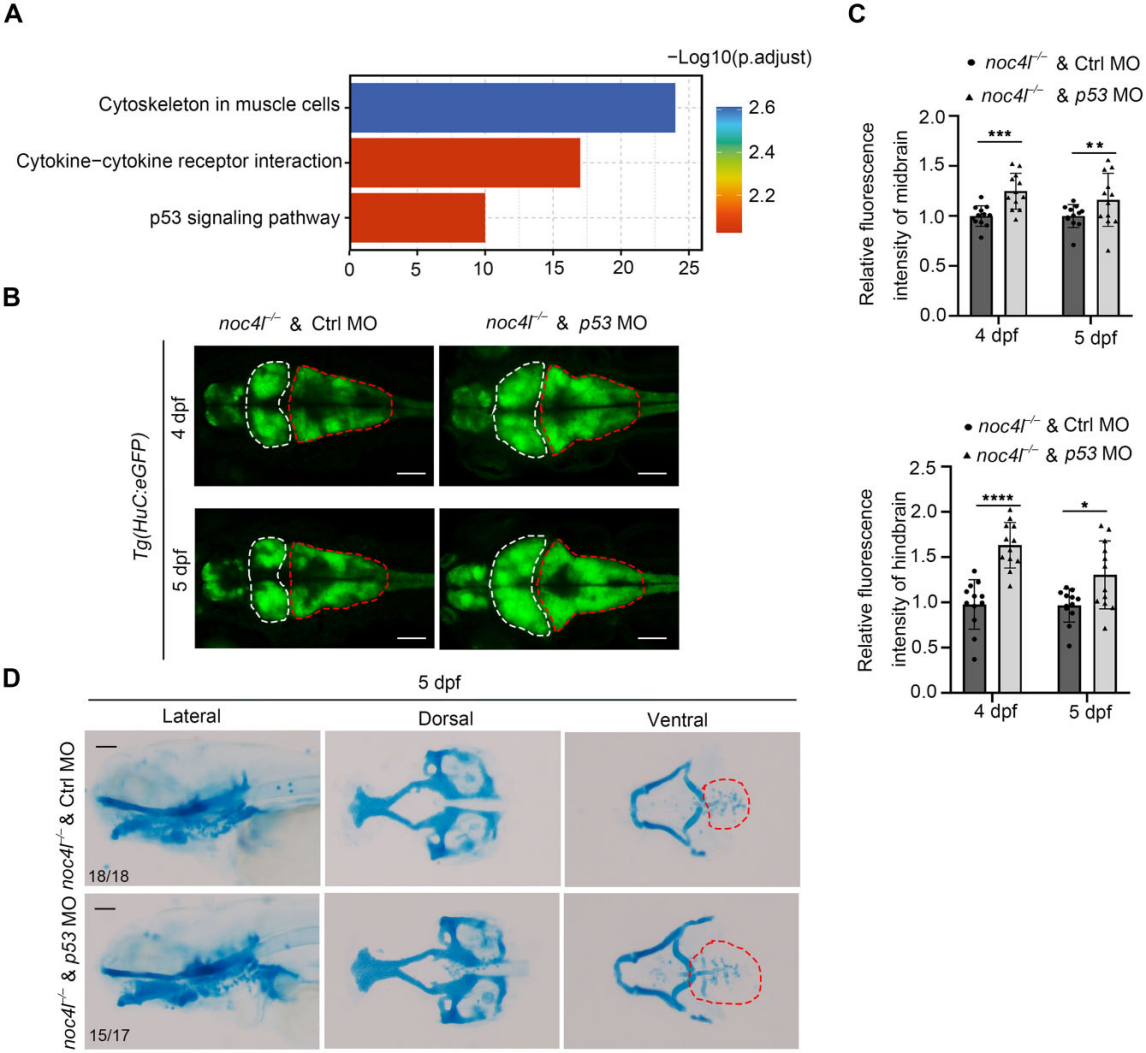
Blocking the p53 signaling pathway for phenotype rescue in *noc4l* mutants. (**A**) KEGG pathway enrichment analysis of upregulated genes in 5 dpf *noc4l* mutant head tissues. (**B**) Neuronal expression in *Tg(HuC:eGFP)* embryos at 4 dpf and 5 dpf following injection of 4 ng *p53* morpholino (MO) or standard control MO (Ctrl MO) at the 1-cell stage. White and red dashed circles indicate the midbrain and hindbrain, respectively. (**C**) Quantification of relative fluorescence intensity in the midbrain and hindbrain at 4 dpf (*n* > 3, ***P < 0.001, ****P < 0.0001) and 5 dpf (*n* > 3, **P < 0.01, *P < 0.05). (**D**) Alcian blue staining of the head region (lateral, dorsal, and ventral views) at 5 dpf following *p53* MO injection, showing rescue of pharyngeal arch cartilage defects. Red dotted circles indicate ceratobranchial cartilage. Scale bar, 50 μm (**B**) or 100 μm (**D**).

The connection between p53 and ribosome assembly is one of the core mechanisms by which cells make life-or-death decisions under stress conditions. While neuronal fluorescence intensity showed significant recovery in the brain, consistent with previous reports of CNS rescue by *p53* knockdown (Azuma et al., 2006), pharyngeal cartilage formation exhibited only marginal improvement (Figure 7B–D), likely because cartilage differentiation is already impaired by 3.5 dpf (Figure 3A and B) and *p53* inhibition cannot restore defective differentiation programs. RNA-seq revealed a marked p53 pathway activation (Figure 7A), suggesting that ribosomal biogenesis defects induce nucleolar stress (Calo et al., 2018). Injecting *p53* MO resulted in minimal phenotypic differences between *noc4l* mutant morphants and controls at 4.5–6.5 dpf (Supplementary Figure S8). Importantly, rosiglitazone-treated *noc4l* mutant morphants showed no difference in apoptosis in the brain and pharyngeal regions compared to controls (Supplementary Figure S9). Since Ribo-seq analysis did not show p53-related changes, the activated p53 signaling likely represents a stress-induced response rather than a translational regulation event. Our results suggest that while both PPARγ and p53 pathways contribute to the phenotypic spectrum in *noc4l* mutants, they likely operate independently rather than through causal relationships.

## Discussion

Defective ribosome biogenesis impairs ribosomal maturation and protein synthesis, leading to multisystem developmental disorders known as ribosomopathies. Despite the universal need for ribosomes, these diseases exhibit tissue-specific pathologies, a phenomenon attributed to translational selectivity (Danilova and Gazda, 2015). NOC4L is part of a nucleolar subcomplex that includes EMG1, NOP14, and UTP14A. EMG1 mutations are known to cause Bowen–Conradi syndrome, which involves psychomotor deficits, microcephaly, and micrognathia (Warda et al., 2016). This prompted us to investigate whether *noc4l* deficiency similarly leads to neurological and pharyngeal cartilage defects.

We compared the expression profiling of *noc4l, nop14*, and *emg1* in zebrafish embryos and observed highly consistent spatiotemporal patterns. At 2 dpf, all three genes were expressed in the midbrain, midbrain–hindbrain boundary, and pharyngeal arches. By 3 dpf, expression expanded to the liver and CHT (Supplementary Figure S1). In *noc4l* mutants, hematopoietic marker analysis showed that *cmyb* expression in the CHT was unchanged at 3 dpf, while by 4 dpf, *cmyb* was moderately downregulated and *rag1* was absent in the thymus (Supplementary Figure S6B). Concurrently, HE staining revealed liver hypoplasia (Supplementary Figure S6A), confirming that *noc4l* deficiency causes hematopoietic and hepatic defects consistent with ribosomopathies.

Kyoto Encyclopedia of Genes and Genomes (KEGG) analysis of Ribo-seq and RNA-seq data showed significant enrichment in metabolic pathways such as drug and retinol metabolism (Figure 5A and B). Ribo-seq identified 12 downregulated genes in the PPAR signaling pathway—all encoding metabolic proteins involved in lipid transport, gluconeogenesis, or bile acid synthesis—with no direct role in ribosome assembly. This indicates that ribosomal dysfunction disrupts metabolic homeostasis. Although PPAR signaling was the sixth most enriched pathway, we selected it as a rescue target due to its established roles in neural and cartilage development. PPARγ is essential for brain and cartilage formation (Monemdjou et al., 2012; Muhsen et al., 2021). PPARγ downregulation inhibits neural progenitor and osteoblast differentiation (Backesjo et al., 2006; Di Giacomo et al., 2017) and promotes chondrocyte apoptosis (Ji et al., 2020). As expected, activating PPARγ with rosiglitazone partially rescued brain and pharyngeal arch malformations in *noc4l* mutants (Figure 6).

TEM revealed fewer normal matrix vesicles in *noc4l* mutants (Figure 5E and H). These extracellular vesicles, secreted by mineralizing cells, are essential for biomineralization, and their loss impairs skeletal development (Anderson et al., 2005; Masi et al., 2015). Although PPARs have not been directly linked to matrix vesicle regulation, they protect chondrocytes by modulating autophagy, apoptosis, inflammation, and MMP activity (Sheng et al., 2023), suggesting that PPARs may protect chondrocytes through these mechanisms.

Ribosomal defects also cause mitochondrial structural and functional impairments (Liu et al., 2024). Mitochondria, as the primary energy source, meet the high demands of ribosome-mediated protein synthesis. PPARγ, a key regulator of mitochondrial function, ameliorates cognitive impairment in Alzheimer’s disease by promoting the clearance of dysfunctional mitochondria through mitophagy (Li et al., 2022). Conversely, PPARγ downregulation causes mitochondrial dysfunction, characterized by the reduced number and swollen morphology of mitochondria (Chiang et al., 2012; Jamwal et al., 2021; He et al., 2023), matching our TEM findings (Figure 5E and F). Poor ER visualization in chondrocytes may further reflect ribosomal and mitochondrial dysfunction.

Metabolic interventions have shown benefit in ribosomopathies, such as growth hormone in cartilage–hair hypoplasia (Obara-Moszynska et al., 2013) and corticosteroids benefiting Diamond–Blackfan anemia patients (Ebert et al., 2005). Although PPARγ has not been targeted in this context, its activation with rosiglitazone partially rescued craniofacial, pharyngeal, and neurodevelopmental defects and extended survival in *noc4l* mutants (Figure 6), consistent with reports that PPARγ upregulation protects against brain defects and chondrocyte apoptosis (Ji et al., 2020; Muhsen et al., 2021).

In summary, we developed a zebrafish model of ribosomopathy and identified PPARγ signaling disruption as a key mechanism underlying head and pharyngeal defects. These insights advance our understanding of ribosome biogenesis and provide a basis for targeting PPARγ in ribosomopathy therapy.

## Materials and methods

### Ethical statement

All animal experiments were conducted in accordance with the requirements of Guangdong Provincial Regulations on the Management of Laboratory Animals.

### Fish strains and maintenance

The AB zebrafish strain was used in this study. To generate *noc4l* mutants, gRNAs targeting the fourth exon were designed and co-injected with Cas9 protein into single-cell stage embryos, as previously described (Chang et al., 2013). Mutant alleles in the F1 generation were identified by PCR amplification and sequencing of the targeted genomic regions, using primers listed in Supplementary Tables S1 and S3. The *Tg(col2a1a:h2afv-mCherry)* line was kindly provided by Dr Chung-Der Hsiao (Chung Yuan Christian University).

### Microinjection and WISH

mRNAs were synthesized *in vitro* using the mMESSAGE mMACHINE™ SP6 kit (AM1340). Microinjection and WISH were performed as described (Jia et al., 2008). cDNA was synthesized from total RNA of 2 dpf embryos. Primers for cloning zebrafish *noc4l* and mouse *Pparg* are provided in Supplementary Table S5. Embryos were fixed overnight at 4°C in 4% paraformaldehyde (PFA). Digoxigenin (DIG)-labeled probes were prepared and used for *in situ* hybridization according to established protocols (Thisse and Thisse, 2014). Target gene fragments were cloned into the pGM-T vector (TIANGEN, VT202) and verified by sequencing; primers are listed in Supplementary Table S4.

### Q-PCR analysis

Total RNA was extracted from 3.5 dpf embryos. cDNA was synthesized using Hifair® III 1st Strand cDNA Synthesis SuperMix (Yeasen, 11202ES08). Q-PCR primers are listed in Supplementary Table S2.

### Paraffin sectioning, HE staining, and IHC staining

Embryos were fixed in 4% PFA at 4°C overnight, washed in phosphate-buffered saline with Tween-20 (PBST), dehydrated, cleared in xylene, and embedded in paraffin. Sections (8 μm) were transferred to slides and baked overnight at 37°C. HE staining was performed as described (Copper et al., 2018). IHC was carried out using a kit from Sangon Biotech (Cat. no. D601037-0050). Puromycin (Solarbio, P8230) was injected at 2 mg/ml. The anti-puromycin antibody (Sigma, MABE342) was used at 1:1000 dilution.

### Alcian blue staining

Embryos at 5 dpf were fixed flat in 4% PFA at 4°C overnight, washed with PBST, and stained with 0.4% Alcian blue solution overnight at room temperature. Embryos were rehydrated, digested with 1% trypsin until transparent, washed again, and stored in glycerol for imaging.

### BrdU labeling

Embryos were incubated in 10 mM BrdU solution (Invitrogen, B23151) on ice for 30 min, then replaced with Holtfreter’s solution, and cultured for 1.5 h. After fixation in 4% PFA and methanol dehydration, embryos were rehydrated, digested with proteinase K, treated with dilute HCl, and blocked with 10% goat serum. Samples were incubated with anti-BrdU (Invitrogen, PA5-32256, 1:1000) at 4°C overnight, followed by Alexa Fluor 488-conjugated secondary antibody (Invitrogen, A11029) and DAPI (Sigma, 10236276001).

### TUNEL assay

Embryos fixed in 4% PFA were dehydrated in methanol and stored at −20°C. After rehydration and proteinase K digestion, samples were post-fixed, blocked with 10% goat serum, and stained with DAPI. Apoptotic cells were detected using the *in situ* cell death detection kit (Roche, 12156792910) according to the manufacturer’s instructions.

### Northern blotting

Total RNA was extracted with TRIzol (Invitrogen, 15596018CN). DIG-labeled probes for 5′ETS, ITS1, and ITS2 were generated by PCR using primers in Supplementary Table S6 and a DIG DNA Labeling Mix (Roche, 11277065910). Northern blotting was performed as described (Lo et al., 2003). Signals were detected with CDP-Star chemiluminescent substrate (Roche, 12041677001).

### Sucrose density gradient centrifugation

This assay was performed as previously described (Liang et al., 2011). Homogenates from 5 dpf embryos were centrifuged at 12000× *g* for 30 min at 4°C. The supernatant was layered onto a 10%–50% sucrose gradient and ultracentrifuged at 38000 rpm for 2 h at 4°C. Fractions were collected, and concentrations were measured and plotted using GraphPad Prism 9.

### Western blotting

Proteins were extracted from 3.5 dpf or 5 dpf embryos using RIPA buffer, separated by SDS–PAGE, and transferred to a nitrocellulose membrane (Cytiva, 10600001). Primary antibodies included anti-puromycin (Sigma, MABE342, 1:1000), anti-PPARγ (Santa Cruz, sc-7196, 1:1000), anti-actin (Affinity, T0022, 1:1000), and anti-tubulin (CWBIO, CW0098M, 1:1000). Horseradish peroxidase-conjugated secondary antibodies (Fude Biotechnology, FDM007/FDR007) were used at 1:1000.

### TEM

Zebrafish heads were dissected and fixed in 2.5% glutaraldehyde/4% PFA at room temperature for 4 h and then at 4°C overnight. Fixed samples were stored on dry ice until sectioning and imaging.

### Drug treatment

For puromycin treatment, 3.5 dpf embryos were injected with 2 mg/ml puromycin and incubated at 29°C for 3 h before western blot or IHC analysis. For PPARγ activation, embryos were treated from 2.5 dpf with 1 μM rosiglitazone (Aladdin, R408369) in Holtfreter’s solution, refreshed daily.

### RNA-seq

Heads from 5 dpf siblings and mutants were collected in TRIzol and stored at −80°C. Sequencing was performed by Gene Denovo Biotechnology on the Illumina Novaseq 6000 platform. Principal component analysis was conducted using the R package gmodels. Differential expression analysis was performed with DESeq2 and edgeR, and gene set enrichment analysis (GSEA) was carried out using GSEA software and MSigDB.

### Ribo-seq

Heads from 5 dpf siblings and mutants were flash-frozen and stored at −80°C before shipment to Gene Denovo Biotechnology. For Ribo-seq, cell suspensions were digested with 0.5% trypsin, treated with cycloheximide, and lysed. Lysates were digested with RNase, and ribosome-protected fragments were isolated by sucrose density gradient ultracentrifugation. RNA was purified for library construction and sequencing.

### Processing of RNA-seq and Ribo-seq data

Genes with fold change ≥2 and *P* < 0.05 in a comparison were considered as significant DEGs. KEGG pathway enrichment analysis was performed on these genes (*P* < 0.05).

### Statistical analysis

All statistical analysis was performed using GraphPad Prism 9. Data are presented as mean ± standard error of the mean (SEM). An unpaired two-tailed Student’s *t*-test was used for comparisons between two groups. Comparisons among more than two groups were performed using one-way ANOVA. ns, not significant, *P* > 0.05; *P < 0.05, **P < 0.01, ***P < 0.001,****P < 0.0001.

## Supplementary Material

mjaf052_Supplemental_File

## Data Availability

RNA-seq data and Ribo-seq data are available at Gene Expression Omnibus under accession number GSE292449.

## Notes

**Author contributions:** T.S.: writing—original draft, investigation. Y.L.: investigation. Y.Z.: investigation. X.L.: investigation. L.Z.: investigation. G.N.: writing—review & editing, project administration. J.Z.: writing—review & editing, project administration.
