# Supplementary material for: NOC4L coordinates neuronal and pharyngeal arch development by regulating ribosome biogenesis
Source: J Mol Cell Biol. 2025 Dec 8;17(12):mjaf052. doi: 10.1093/jmcb/mjaf052 (PMC13285729; doi:10.1093/jmcb/mjaf052)
Supplement: mjaf052_Supplemental_File [file mjaf052_supplemental_file.pdf]

Supplementary material

Supplementary Figures

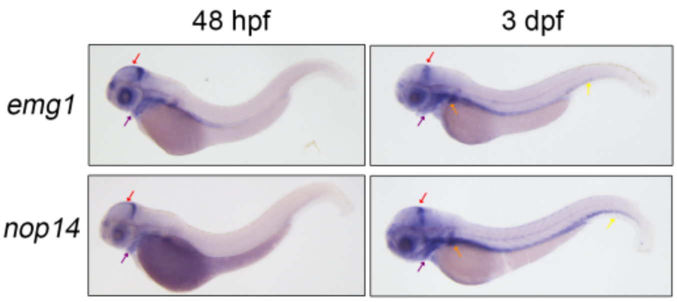

Supplementary Figure S1. Expression patterns of *emg1* and *nop14* in zebrafish at 48 hpf and 3 dpf as detected by WISH. The red arrow indicates the midbrain-hindbrain boundary of the embryo. The purple arrow indicates the pharyngeal arches of the embryo. The orange arrow indicates the liver of the embryo. The yellow arrow indicates the caudal hematopoietic tissue of the embryo. hpf, hours post-fertilization. dpf, days post-fertilization. WISH, whole-mount *in situ* hybridization.

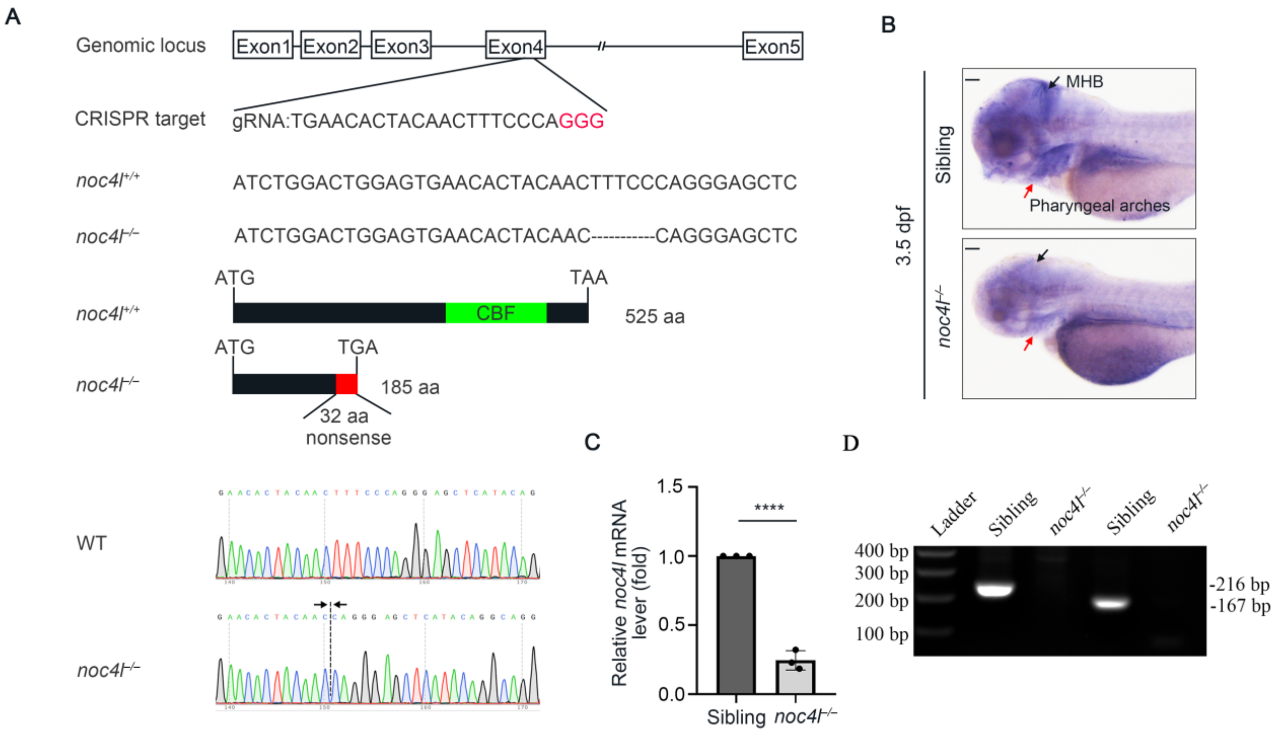

Supplementary Figure S2. Construction of *noc4l* mutants using CRISPR/Cas9 gene editing technology. (**A**) Schematic diagram of the target design and mutation results for *noc4l* gene editing. A CRISPR/Cas9 target was designed in the fourth exon region, and a mutant with a 5-base pair deletion was successfully obtained through gene editing experiments and sequencing analysis. This mutation resulted in premature termination of protein translation, leading to the loss of the critical functional domain CBF and thus rendering the protein nonfunctional. (**B**) Validation of successful *noc4l* mutant construction by WISH. MHB (midbrain hindbrain boundary). (**C**) Zebrafish embryos at 3.5 dpf were collected for RNA extraction and cDNA synthesis, followed by qPCR quantification of relative *noc4l* mRNA levels in sibling versus *noc4l* mutant embryos. Data are expressed as mean ± SEM (n = 3, ****$P < 0.0001$). dpf, days post-fertilization. (**D**) Genotyping PCR was performed to distinguish between siblings and mutants. The 3' ends of the mutF and mutR primers were designed to contain the 5-base deletion sequence. The primer sequences are provided in Supplementary Table S3. Genotypic analysis of siblings revealed amplification products of 216 bp and 167 bp using primer sets *noc4l* F/mutR and *noc4l* mutF/R, respectively. No amplification product was observed in the mutants.

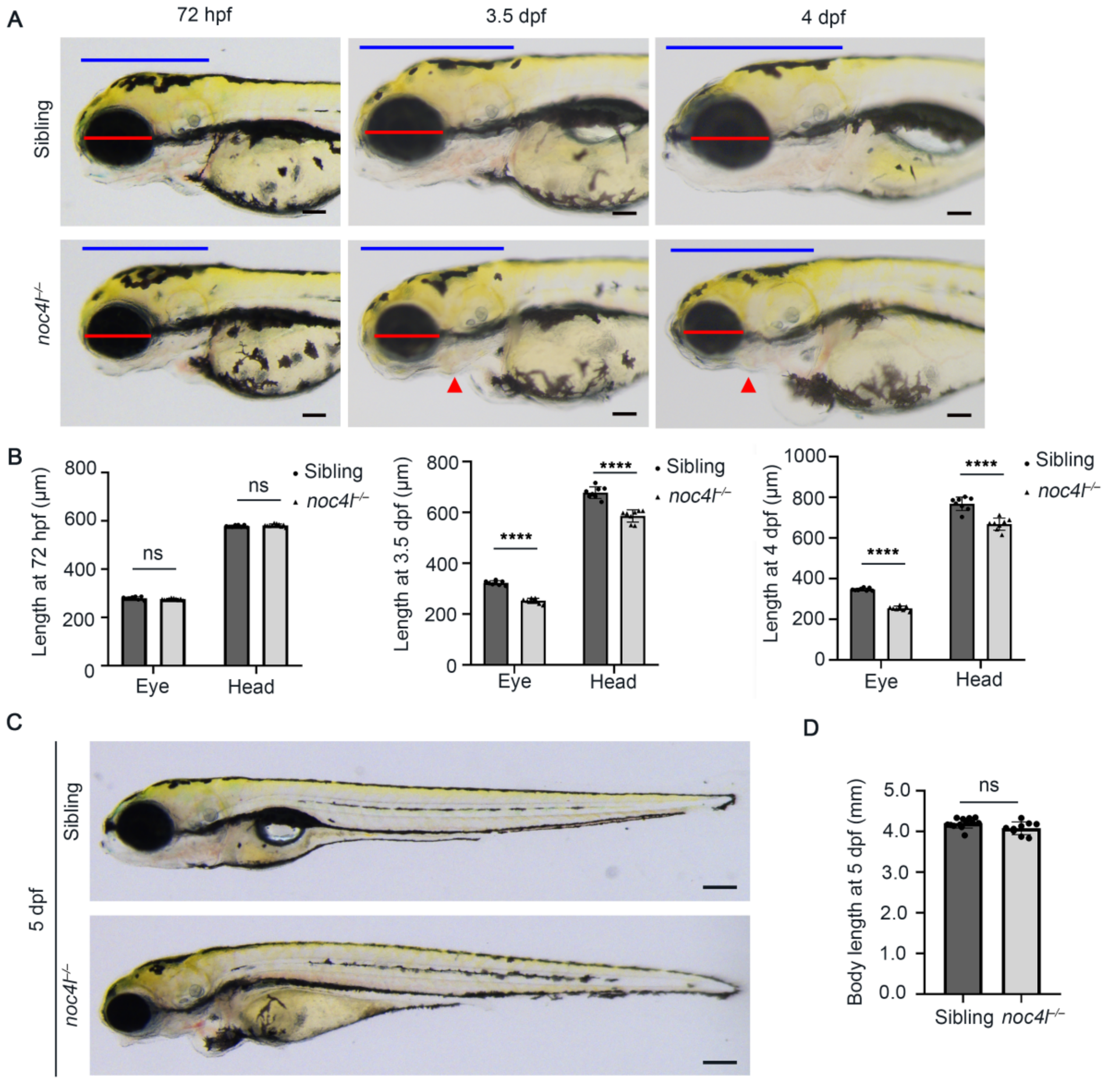

Supplementary Figure S3. (**A**) Bright-field images show that *noc4l* mutants at 3.5 dpf and 4 dpf exhibit reduced head size, smaller eyes, underdeveloped mandibular structures, pericardial edema, and absence of swim bladder. All samples are shown in lateral view. The blue dashed line indicates embryonic head size, and the red dashed line indicates eye size. Scale bar, 100 μm. (**B**) Statistical analysis of head size in embryos at 3 dpf ($n > 3$, ns), 3.5 dpf ($n > 3$, ****$P < 0.0001$), and 4 dpf ($n > 3$, ****$P < 0.0001$), and eye size in embryos at 3 dpf ($n > 3$, ns), 3.5 dpf ($n > 3$, ****$P < 0.0001$), and 4 dpf ($n > 3$, ****$P < 0.0001$). (**C**) Comparison of body length between siblings and *noc4l* mutants at 5 dpf. (**D**) Statistical analysis of body length comparison between siblings and *noc4l* mutants at 5 dpf ($n > 3$, ns). DV, dorsal view; LV, lateral view. ns indicates no significant difference, $P > 0.05$.

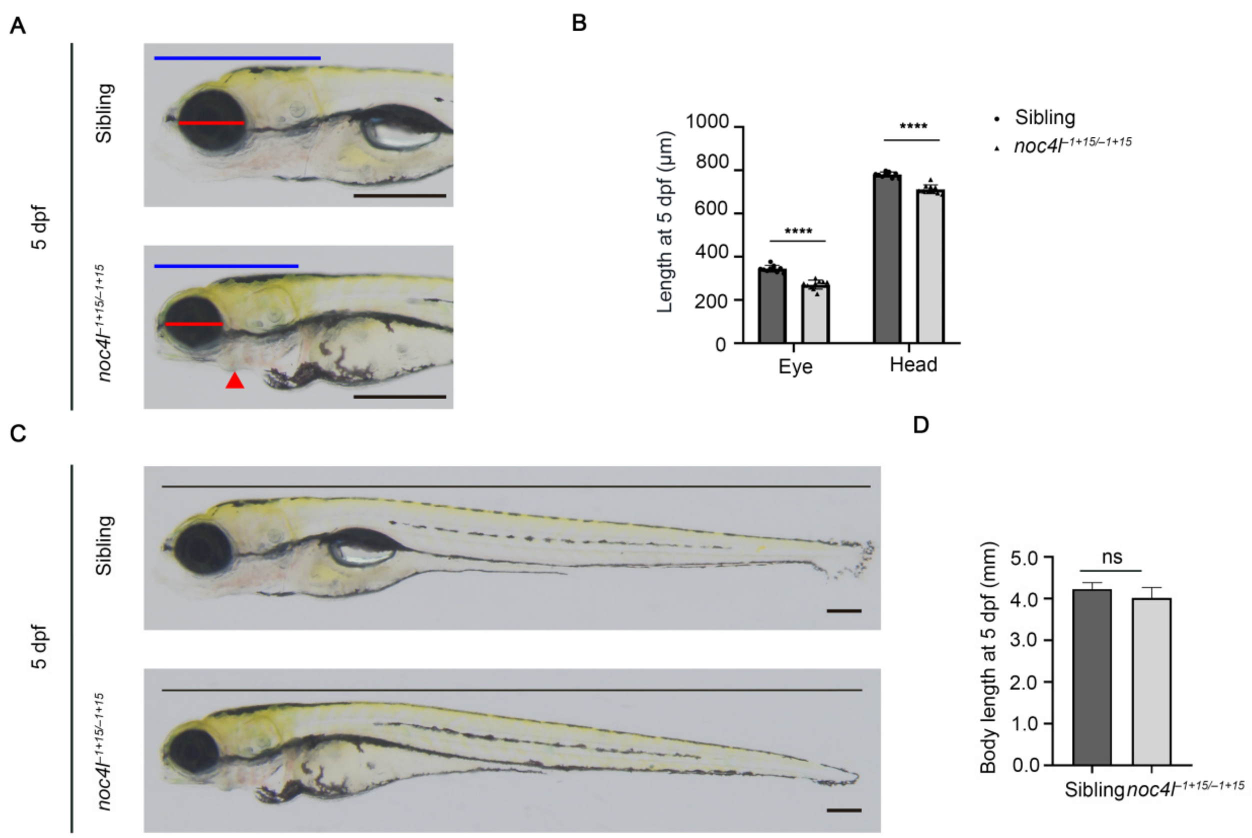

Supplementary Figure S4. Phenotypic analysis of an alternative *noc4l* mutant genotype in zebrafish. (**A**) Bright-field images show that the *noc4l* $^{-1+15/-1+15}$ mutants exhibited reduced head size, smaller eyes, diminished mandibular structures, pericardial edema, and an absence of swim bladder at 5 dpf. All embryos are shown in lateral view. The blue line indicates the head size of the embryos. The red line indicates the eye size of the embryos. (**B**) The panel shows the statistical analysis of the head size ($n > 3$, ****$P < 0.0001$) and the eye size ($n > 3$, ****$P < 0.0001$) of the embryos at 5 dpf. (**C**) Comparison of body length between siblings and *noc4l* $^{-1+15/-1+15}$ mutants at 5 dpf. (**D**) Statistical analysis of body length comparison between siblings and *noc4l* $^{-1+15/-1+15}$ mutants at 5 dpf ($n > 3$, ns). ns indicates no significant difference, $P > 0.05$.

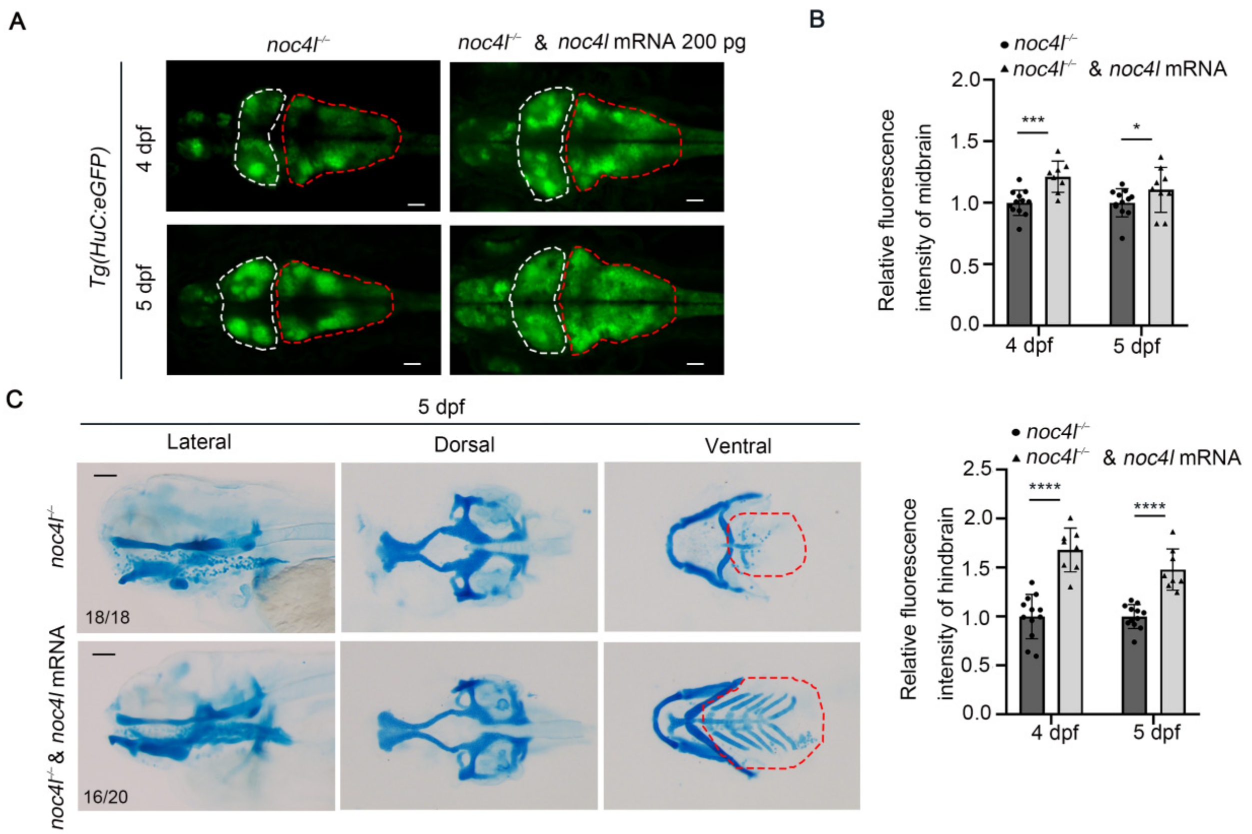

Supplementary Figure S5. (**A**) Fluorescence imaging results of embryos at 4 dpf and 5 dpf in the *Tg(HuC:eGFP)* background (following microinjection of *noc4l* mRNA (200 pg/nl, 1 nl/embryo) into the yolk sac of 1-cell stage zebrafish embryos to rescue phenotypic defects in *noc4l* mutants). The midbrain region is outlined by a white dashed circle, and the hindbrain region by a red dashed circle. Scale bar, 50 μm. (**B**) Statistical analysis of relative fluorescence intensity in the midbrain ($n > 3$, ***$P < 0.001$) and hindbrain ($n > 3$, ****$P < 0.0001$) of 4 dpf embryos, as well as in the midbrain ($n > 3$, *$P < 0.05$) and hindbrain ($n > 3$, ****$P < 0.0001$) of 5 dpf embryos. (**C**) Alcian blue staining images of 5 dpf embryos, following microinjection of *noc4l* mRNA (200 pg/nl, 1 nl/embryo) into the yolk sac of 1-cell-stage zebrafish embryos to rescue *noc4l*-mutant phenotypes. Images show the head regions of siblings and *noc4l* mutants at 5 dpf (lateral, dorsal and ventral views), the ceratobranchial cartilage is indicated by red dotted circle. Scale bar, 100 μm.

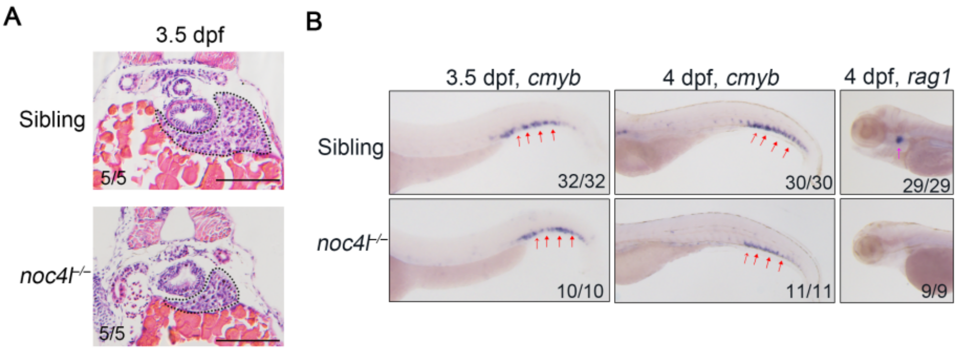

Supplementary Figure S6. (**A**) Hepatic structures of wild-type (upper panel) and *noc4l* mutants (lower panel) at 3.5 dpf displayed by HE staining of paraffin sections. Black dashed lines indicate liver size. Scale bar, 100 μm. (**B**) Expression changes of *cymb* and *rag1* in *noc4l* mutants embryos at 4 dpf compared with their siblings. The red arrow indicates the caudal hematopoietic tissue. The pink arrow indicates the thymus.

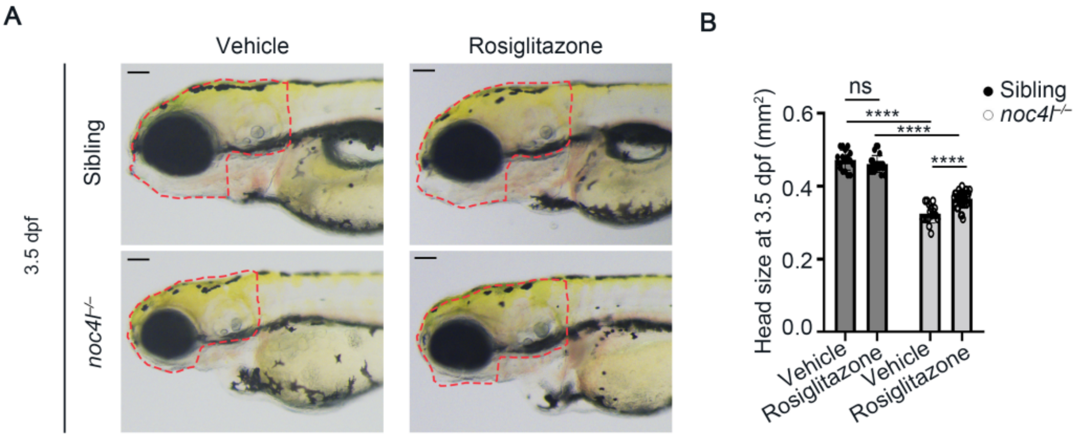

Supplementary Figure S7. (**A**) The results of bright-field imaging of the embryos at 3.5 dpf after incubating embryos at 2.5 dpf overnight in Holtfreter's solution containing rosiglitazone or DMSO (lateral view). Location of head is indicated by red dotted circle. (**B**) The statistical analysis of head size in 3.5 dpf embryos following drug treatment ($n > 3$, ns, $^{****}P < 0.0001$). ns indicates no significant difference, $P > 0.05$.

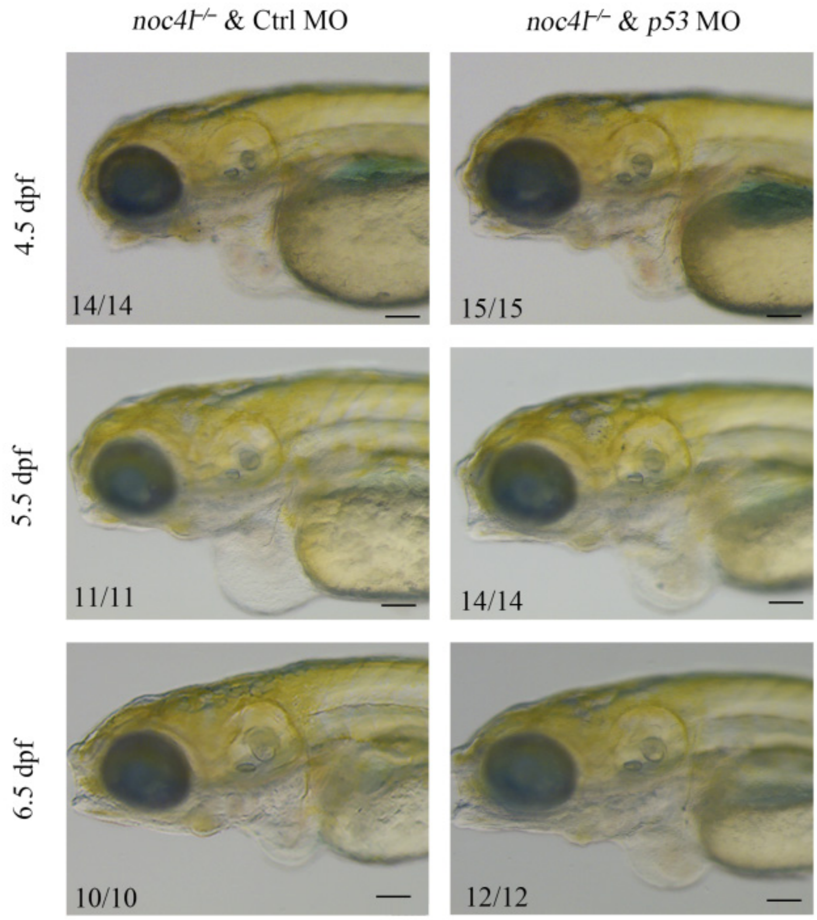

Supplementary Figure S8. Bright-field images of 4.5 dpf, 5.5 dpf and 6.5 dpf embryos (following microinjection of p53 MO into the yolk sac of 1-cell stage zebrafish embryos to rescue phenotypic defects in *noc4l* mutants). All samples are shown in lateral view. Scale bar, 100 μm.

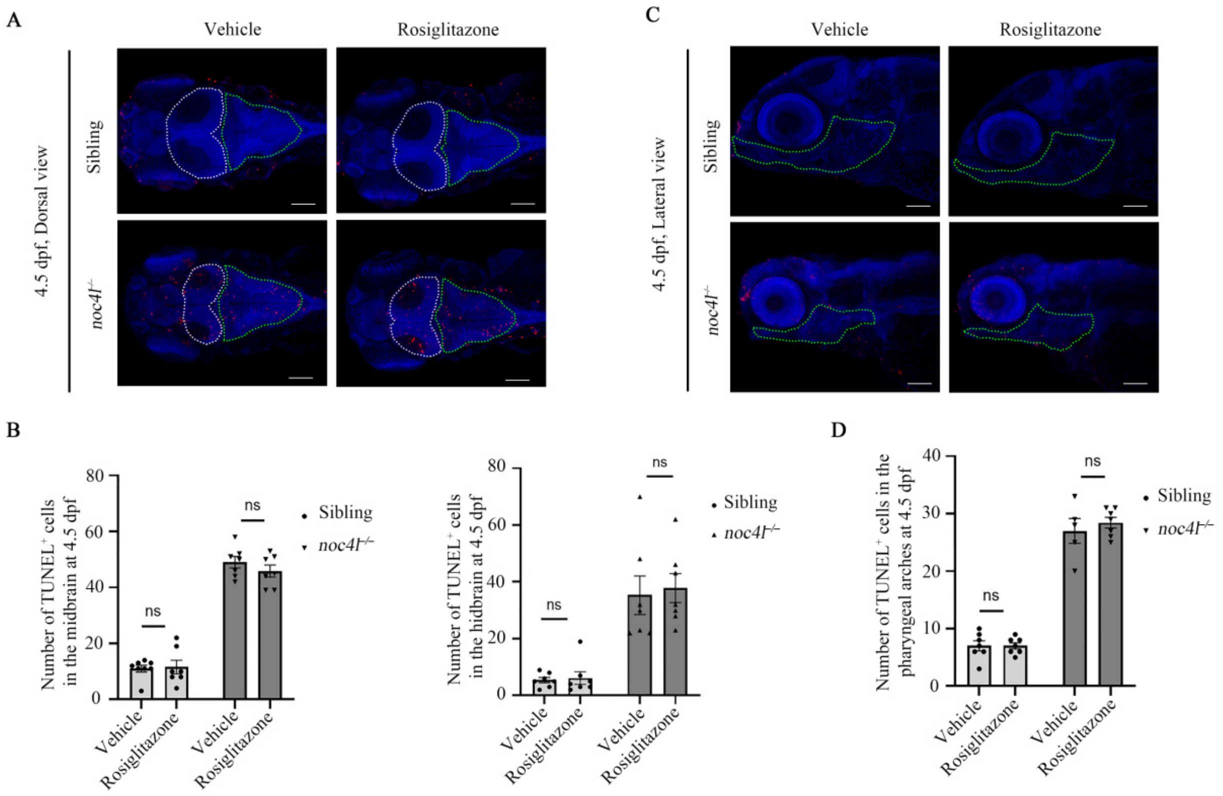

Supplementary Figure S9. Analyses of cell apoptosis in midbrain, hindbrain and pharyngeal arches: comparison between control and rosiglitazone-treated groups. (**A**) Fluorescence imaging of 4.5 dpf embryos after incubating 2.5 dpf embryos for 2 days in Holtfreter's solution containing rosiglitazone or DMSO (with solution change every 24 h). TUNEL assay (red signal) shows apoptotic cells in the midbrain and hindbrain of control and rosiglitazone-treated groups (dorsal view). The midbrain region is indicated by a white dotted circle, while the hindbrain region is marked by a green dotted circle. (**B**) Statistical analysis of apoptotic cells in the midbrain and hindbrain ($n > 3$, ns). (**C**) TUNEL assay (red signal) detecting apoptotic cells in the pharyngeal arches of control and rosiglitazone-treated groups (lateral view). The pharyngeal arches region is outlined by a green dotted circle. (D) Statistical analysis of apoptotic cells in the pharyngeal arches ($n > 3$, ns). Scale bar, 100 μm. ns indicates no significant difference, $P > 0.05$.

Supplementary Tables

Supplementary Table S1. The gRNA sequence for *noc4l* gene.

Name	gRNA
noc4l	GTGAACACTACAACTTTCCCA

Supplementary Table S2. The primers for Q-PCR.

Name	Primer
noc4l F	5’-CTTTCCAAGTCAAGGAGACATCGCT-3’
noc4l R	5’-GTATCGCACTGAACTCATGACGTAG-3’

Supplementary Table S3. The genotyping primers for PCR.

Name	Primer
noc4l F	5’-AGGAAGTGTCTCTTTGCCATATGTA-3’
noc4l R	5’-AAATGTACAACATAAAGTACGTTGG-3’
noc4l mutF	5’-GGACTGGAGTGAACACTACAACTTTCC-3’
noc4l mutR	5’-CAGGGCCTGTATGAGCTCCCTGGGAAA-3’

Supplementary Table S4. The primers for WISH.

Name	Primer
noc4l F	5’-ACATTCCCAACCAAGCATCA-3’
noc4l R	5’-CCTTCAGTTCTCGCTCCATT-3’
emg1 F	5’-GCGTCTTGTCGTTGTTTTAGAAGGA-3’
emg1 R	5’-AATGAGTCTTAACAAACGCTGAGGG-3’
nop14 F	5’-ATCGTGCAGATTCTGGATTATGGGA-3’
nop14 R	5’-TCAACAAAACACTTCAACAGGTCTG-3’

Supplementary Table S5. The primers for cloning zebrafish *noc4l* and mouse *Pparg*.

**Name**	**Primer**
noc4l F	5'-AACGAATTCATGGCGCCGTCCGGGGACAGCAACGTGAAA-3'
noc4l R	5'-CTAATCGATCTCTAGAGTAAAGTGTACTCCGAGCACCTC-3'
Pparg F	5'-TCTATCGATATGGTTGACACAGAGATGCCATTCT-3'
Pparg R	5'-AACCTCGAGCTAATACAAGTCCTTGTAGATCTCC-3'

Supplementary Table S6. The primers for northern blotting.

**Name**	**Primer**
5'ETS probe F	5'-CCGGTCTACCTCGAAAGTC-3'
5'ETS probe R	5'-CGAGCAGAGTGGTAGAGGAAG-3'
ITS1 probe F	5'-CTCGGAAAACGGTGAACCTG-3'
ITS1 probe R	5'-GTGTTCGTTTCAGGGTCCG-3'
ITS2 probe F	5'-CCTAAGCGCAGACCGTCAC-3'
ITS2 probe R	5'-AGCGCTGGCCTCGGAGATC-3'
